# Silent threat: how preoperative albumin levels affect geriatric cancer surgery outcomes?

**DOI:** 10.1186/s12893-026-03659-9

**Published:** 2026-03-16

**Authors:** Mustafa Kemal Sahin

**Affiliations:** Department of Anesthesiology, Dr. Abdurrahman Yurtaslan Oncology Training and Research Hospital, Ankara, Turkey

**Keywords:** Hypoalbuminemia, Aged, Neoplasms, Surgical Procedures, Operative, Postoperative Complications, Serum Albumin

## Abstract

**Background:**

Preoperative hypoalbuminemia is a common condition in elderly cancer patients and may adversely affect surgical outcomes. This study aimed to evaluate the association between preoperative serum albumin levels and postoperative complications, length of hospital stay, and mortality in geriatric patients undergoing cancer surgery.

**Methods:**

This retrospective single-center study included 330 patients aged 65 years and older who underwent cancer surgery between 2018 and 2024. Patients were divided into two groups based on preoperative albumin levels: hypoalbuminemia (< 3.5 g/dL) and normal (≥ 3.5 g/dL). Demographic data, comorbidities, surgical details, and postoperative outcomes were compared between groups.

**Results:**

Of the patients, 46% had hypoalbuminemia. Patients with low albumin were significantly older and more likely to be functionally dependent. The incidence of postoperative pulmonary complications (PPCs) was higher in the hypoalbuminemia group (28% vs. 9%, *p* = 0.002). Furthermore, these patients had longer ICU and hospital stays and higher 30-day mortality (11% vs. 5%, *p* = 0.003). Each 1 g/dL decrease in preoperative albumin level was independently associated with a 3.49-fold increase in the odds of PPCs (OR 3.49, 95% CI 1.17–10.44). Advanced age and higher ASA class were also associated with prolonged hospital stay, while lower hemoglobin and current smoking were independent risk factors for PPCs.

**Conclusion:**

Preoperative hypoalbuminemia is a significant and independent predictor of adverse postoperative outcomes in geriatric cancer patients. Preoperative serum albumin may serve as a valuable marker of physiological reserve and perioperative risk stratification in geriatric cancer patients.

**Trial registration:**

ClinicalTrials.gov, NCT06998030 (registered on 13 May 2025; retrospectively registered).

## Introduction

As the global population ages, the number of geriatric patients diagnosed with cancer continues to rise. Surgery remains a cornerstone of cancer treatment, yet elderly patients undergoing oncologic surgery face unique physiological and clinical challenges. Age-related decline in physiological reserves, increased prevalence of comorbidities, and reduced tolerance to surgical stress contribute to heightened perioperative risks in this population [1]. Identifying reliable preoperative risk factors is crucial for optimizing surgical outcomes and improving perioperative management strategies in geriatric cancer patients.

Among the various biomarkers used for preoperative risk assessment, serum albumin levels have gained increasing attention due to their strong association with surgical outcomes. Albumin, a key protein synthesized by the liver, plays a vital role in maintaining oncotic pressure, modulating inflammatory responses, and supporting tissue repair [2]. Hypoalbuminemia—defined as low serum albumin levels—has historically been associated with malnutrition; however, it is increasingly recognized as a multifactorial biomarker reflecting not only nutritional status but also systemic inflammation, disease burden, and impaired physiological reserve [3]. Numerous studies have linked hypoalbuminemia to increased rates of postoperative complications, prolonged hospital stays, and higher mortality across various surgical disciplines [1, 4]. For instance, in robotic-assisted pulmonary lobectomy, patients with preoperative albumin levels < 3.5 g/dL experienced significantly higher complications (44.2% vs. 24.9%) and reduced long-term survival [4]. Similarly, in bariatric surgery, hypoalbuminemia (< 3.5 g/dL) was associated with a 42% increased risk of 30-day mortality and prolonged hospitalization [3].

Despite the well-established prognostic value of albumin in general surgical populations, its role in geriatric oncologic surgery remains insufficiently explored. Cancer itself is a hypermetabolic state that exacerbates protein depletion, and elderly patients are particularly vulnerable to malnutrition and sarcopenia [2]. These factors further compound the potential impact of hypoalbuminemia on postoperative recovery and survival. Notably, preoperative hypoalbuminemia in cancer patients correlates with larger tumor sizes and advanced disease stages, reflecting the interplay between systemic inflammation and cancer progression [4]. Furthermore, the CRP/albumin ratio—a combined marker of inflammation and nutritional status—has emerged as a predictor of postoperative delirium and mortality in elderly surgical populations [2]. This highlights the need to evaluate albumin not merely as a nutritional marker but as a holistic indicator of physiological resilience in geriatric cancer patients.

This study aims to fill this gap by investigating the association between preoperative serum albumin levels and postoperative outcomes in geriatric cancer patients. By analyzing real-world clinical data, we seek to determine whether albumin can serve as a reliable biomarker for surgical risk stratification and whether it should be more routinely integrated into preoperative assessment protocols. A better understanding of this relationship may pave the way for targeted perioperative interventions, such as nutritional optimization or anti-inflammatory therapies, to improve surgical outcomes in this vulnerable patient population.

## Materials and methods

This retrospective study was approved by the Institutional Review Board of Dr. Abdurrahman Yurtaslan Oncology Training and Research Hospital (IRB Number: 2025–03/53) and registered at ClinicalTrials.gov (NCT06998030) on 13 May 2025. The study was conducted in accordance with the ethical principles of the Declaration of Helsinki. The requirement for informed consent was waived due to the retrospective nature of the study and anonymization of patient data.

Inclusion criteria were age ≥ 65 years, confirmed cancer diagnosis with surgical intervention, and availability of preoperative albumin measurement. Exclusion criteria included preoperative sepsis, severe infection, active inflammatory disease, palliative or emergency surgery, incomplete data, or insufficient follow-up. Incomplete records were defined as missing essential information for primary outcomes, such as postoperative complications or 30-day mortality. Insufficient follow-up was defined as lack of documented follow-up data for at least 30 days after surgery.

Patients were grouped by preoperative albumin level (< 3.5 g/dL vs. ≥ 3.5 g/dL) and compared for postoperative complications, hospital and ICU stay, and 30-day mortality. In addition to the categorical analysis, albumin was also evaluated as a continuous variable (per 1 g/dL change) in a separate multivariate logistic regression model to quantify the incremental effect on PPC risk. Postoperative pulmonary complications were defined and classified according to the European Perioperative Clinical Outcome (EPCO) criteria [5]. Preoperative serum albumin levels were measured using the standard bromocresol green (BCG) colorimetric assay. According to hospital protocol, preoperative blood samples are routinely collected from inpatients after overnight fasting. Preoperative hypoalbuminemia was defined as a serum albumin concentration of less than 3.5 g/dL. In accordance with standard clinical practice, preoperative albumin levels were obtained from medical records, with the measurement closest to the date of surgery—within one week prior to the operation—considered for analysis. Demographic, clinical, and perioperative data were collected and anonymized in accordance with ethical standards.

### Potential biases

As a retrospective, single-center study, there is a risk of selection and information bias, as well as limited generalizability. Uncontrolled confounders such as cancer stage, tumor burden, and surgical technique may have affected results. To reduce bias, strict inclusion and exclusion criteria and a standardized data collection protocol were used.

### Statistical analysis

A power analysis was performed prior to data collection, indicating that a minimum of 280 patients would be required to achieve 90% power to detect a clinically significant difference in postoperative complication rates between groups (α = 0.05). To increase the robustness of the study and account for potential exclusions, a total of 330 patients aged 65 years and older who underwent cancer surgery between 2018 and 2024 and had documented preoperative albumin levels were included.

All statistical analyses were performed using IBM SPSS Statistics version 25. Continuous variables were assessed for normality using the Kolmogorov–Smirnov test. Data are presented as mean ± standard deviation or median (minimum–maximum) for continuous variables, and as numbers and percentages for categorical variables. Comparisons between groups were conducted using the independent samples t-test or Mann–Whitney U test, as appropriate. Categorical variables were compared using the Chi-square or Fisher’s exact test.

To explore the relationship between preoperative albumin levels and clinical outcomes, patients were categorized into two groups according to serum albumin concentration (< 3.5 g/dL vs. ≥ 3.5 g/dL). Demographic characteristics, comorbidities, cancer diagnoses, surgical procedures, and perioperative variables were compared between these groups. Associations between albumin levels and postoperative pulmonary complications, intensive care unit and hospital length of stay, and 30-day mortality were evaluated.

Generalized linear models were used to analyze hospital length of stay. To address potential confounding related to disease severity and procedural complexity, ASA physical status was used as a surrogate marker of overall disease burden and physiological reserve, while surgical duration (≥ 3 h) and upper abdominal incision were included as proxies for procedural complexity and surgical stress. Covariates were selected based on clinical relevance rather than automated stepwise procedures to ensure consistency across multivariate models and to minimize overfitting.

Accordingly, preoperative albumin, age, ASA class, hemoglobin level, functional status (independent vs. dependent), upper abdominal incision, surgical duration, and recent respiratory tract infection were entered simultaneously into the multivariate models to evaluate the independent association between albumin and postoperative outcomes.

Univariate generalized linear models were performed as exploratory analyses. Multivariate logistic regression analysis was used to identify independent predictors of postoperative pulmonary complications. Odds ratios (ORs) and 95% confidence intervals (CIs) were reported. A two-sided *p*-value < 0.05 was considered statistically significant.

## Results

A total of 330 geriatric cancer patients who underwent surgery were included in the analysis. Of these, 152 patients (46%) had preoperative serum albumin levels below 3.5 g/dL, while 178 patients (54%) had albumin levels of 3.5 g/dL or higher.

As shown in Table 1, patients with low albumin were significantly older than those with normal albumin levels (mean age 73 vs. 70 years, *p* < 0.001). The proportion of male patients was higher in the normal albumin group (62% vs. 38%, *p* = 0.006). Most patients were classified as ASA III (71%), with no significant difference in ASA distribution between the groups (*p* = 0.147). Body mass index (BMI) categories were similar across groups (*p* = 0.096). While the majority of patients were functionally independent, partial or full dependency was more common among those with low albumin (*p* = 0.002). Smoking status did not differ significantly between groups (*p* = 0.254).

**Table 1:** Demographic data

Patient Characteristics, n (%)	Albumin < 3.5 g/dl *n* = 152 (46)	Albumin ≥ 3.5 g/dl *n* = 178 (54)	Total *n* = 330 (100)	*p*-value
Age (years), Mean ± SD	73 ± 5	70 ± 5		0.000*
Gender, n (%)				**0.006**^**†**^
Male	76 (38)	126 (62)	202 (61)	
Female	76 (59)	52 (41)	128 (39)	
ASA, n (%)				0.147^†^
II	26 (34)	50 (66)	76 (23)	
III	114 (48)	122 (52)	236 (71)	
IV	12 (67)	6 (33)	18 (6)	
BMI*, kg/m2, n (%)				0.096^†^
0–20 (kg/m^2^)	18 (60)	12 (40)	30 (9)	
21–35 (kg/m^2^)	132 (46)	152 (54)	284 (86)	
35 (kg/m^2^)	2 (13)	14 (87)	16 (5)	
History of chemotherapy, n (%)	32 (46)	78 (54)	70 (100)	0.963^†^
History of radiotherapy, n (%)	14 (35)	26 (35)	40 (100)	0.285^†^
Functional Status, n (%)				**0.002**^**†**^
Independent	104 (39)	160 (61)	264 (80)	
Partially Dependent	44 (71)	18 (29)	62 (19)	
Dependent	4 (0)	0 (100)	4 (1)	
Smoke Status, n (%)				0.254^†^
Never Smoked	92 (50)	92 (50)	184 (56)	
Smoker	60 (41)	86 (59)	146 (44)	

Values are presented as mean ± SD, n: Number of patients, %: Column Percentage

Bold values indicate statistical significance (*p* < 0.05)

*SD* Standard Deviation, *BMI* Body Mass Index, *ASA* American Society of Anesthesiologists Classification

*Mann-witney u, †Chi-square test

The prevalence of comorbidities such as hypertension, coronary artery disease, COPD, and diabetes was similar between the two groups (all *p* > 0.05, Table 2). Regarding surgical procedures, colectomy (46%) and gastrectomy (22%) were the most frequently performed operations (Table 3). Hypoalbuminemia was more common among patients undergoing gastrectomy (*p* = 0.001), hyperthermic intraperitoneal chemotherapy (*p* = 0.043), and Whipple procedures (*p* = 0.023). Preoperative pulmonary function status was similar between groups, although dyspnea at rest or persistent dyspnea was observed only in the low albumin group (*p* = 0.201) (Table 4).

**Table 2:** The relationship between preoperative comorbidities and albumin levels

Pre-Operative Co-Morbidities	Albumin < 3.5 g/dl *n* = 152 (100)	Albumin ≥ 3.5 g/dl *n* = 178 (100)	Total *n* = 330 (100)	*p* value
None, n (%)	24 (16)	50 (28)	74 (22)	0.026*
Hypertension, n (%)	52 (34)	52 (29)	104 (32)	0.453*
Coronary artery disease (CAD), n (%)	14 (9)	20 (11)	34 (10)	0.606*
Asthma, n (%)	6 (4)	10 (6)	16 (5)	0.708*
Chronic obstructive pulmonary disease (COPD), n (%)	24 (16)	20 (11)	44 (13)	0.305*
Diabetes (DM), n (%)	22 (15)	22 (12)	44 (13)	0.589*
Chronic kidney disease (CKD), n (%)	2 (1)	0 (0)	2 (1)	–
Psychiatric disorder, n (%)	2 (1)	0 (0)	2 (1)	–
Neurological disorder, n (%)	6 (4)	2 (1)	8 (2)	0.086*
Hematological disorder (Blood disorder), n (%)	0 (0)	2 (1)	2 (1)	–

n: Number of patients, %: Column Percentage

Bold values indicate statistical significance (*p* < 0.05)

*Chi-square test

**Table 3:** Surgical procedure name and albumin levels

Surgical procedure name	Albumin < 3.5 g/dl *n* = 152 (100)	Albumin ≥ 3.5 g/dl *n* = 178 (100)	Total *n* = 330	*P* value
Esophagectomy, n (%)	2 (1)	6 (3)	8 (2)	0.356*
Gastrectomy, n (%)	50 (33)	24 (14)	74 (22)	0.001*
Enterectomy, n (%)	2 (1)	0 (0)	2 (1)	–
Colectomy, n (%)	64 (42)	86 (48)	150 (46)	0.056*
Hepatectomy, n (%)	0 (0)	6 (3)	6 (2)	0.046*
Hyperthermic Intraperitoneal Chemotherapy, n (%)	6 (4)	2 (1)	8 (2)	0.043*
Whipple procedure, n (%)	8 (5)	2 (1)	10 (3)	0.023*
Nephrectomy, n (%)	4 (3)	4 (2)	8 (2)	0.758*
Prostatectomy, n (%)	0 (0)	22 (12)	22 (7)	0.000*
Cystectomy, n (%)	2 (1)	2 (1)	4 (1)	–
Debulking, n (%)	14 (9)	24 (14)	38 (12)	0.026*

n: Number of patients, %: Column Percentage

Bold values indicate statistical significance (*p* < 0.05)

*Chi-square test

**Table 4:** Association of albumin levels with intraoperative variables and patients’ pulmonary function status

		Albumin < 3.5 g/dl	Albumin ≥ 3.5 g/dl	Total	p
Surgical incision site	Upper abdominal incision	78 (51)	48 (27)	126 (38)	0.003*
	Lower abdominal incision	30 (20)	68 (38)	98 (30)	
	Laparoscopic	44 (29)	62 (35)	106 (32)	
	Total, n (%)	152 (100)	178 (100)	330 (100)	
Operation time	< 2 h	14 (9)	14 (8)	28 (8)	0.769*
	2–3 h	88 (58)	96 (54)	184 (56)	
	More than 3 h	50 (33)	68 (38)	118 (36)	
	Total, n (%)	152 (100)	178 (100)	330 (100)	
Type of surgery	Open surgery	108 (71)	116 (65)	224 (68)	0.420*
	Laparoscopic surgery	44 (29)	62 (35)	106 (32)	
	Total, n (%)	152 (100)	178 (100)	330 (100)	
Patient's pulmonary function status	No dyspnea, n (%)	94 (62)	128 (72)	222 (67)	0.201^†^
	Dyspnea with exertion, n (%)	52 (34)	50 (28)	102 (31)	
	Dyspnea at rest, n (%)	4 (3)	0 (0)	4 (1)	
	Dyspnea all the time, n (%)	2 (1)	0 (0)	2 (1)	

*n* Number of patients, %: Column Percentage

Bold values indicate statistical significance (*p* < 0.05)

*Chi-square test, ^**†**^ Kruskal Wallis

Upper abdominal incisions were significantly more frequent in the low albumin group (51% vs. 27%, *p* = 0.003, Table 4), while operation time and surgical approach (open vs. laparoscopic) did not differ significantly between groups (*p* = 0.769 and *p* = 0.420, respectively).

Postoperative pulmonary complications (PPCs) occurred in 58 patients (18%), with a significantly higher incidence in the low albumin group (28% vs. 9%, *p* = 0.002, Table 5). Among specific complications such as pleural effusion (*p* < 0.001), atelectasis (*p* = 0.016), pulmonary embolism (*p* = 0.023), and emergency re-intubation (*p* = 0.016) were also more frequent among patients with low albumin (Table 6). Patients with low albumin had longer postoperative ICU stays (mean 29.6 ± 27.0 h, Q1: 11, Q3: 47) and hospital stays (mean 10.3 ± 5.3 days, Q1: 6, Q3: 14), compared to patients with normal albumin (mean 25.7 ± 30.0 h, Q1: 5, Q3: 45 for ICU; mean 8.4 ± 6.0 days, Q1: 4, Q3: 12 for hospital stay), as shown in Table 7. Thirty-day mortality was significantly higher in the low albumin group (11% vs. 5%, *p* = 0.003, Table 5).

**Table 5:** Association of albumin levels with postoperative pulmonary complications and 30-day mortality

		Albumin < 3.5 g/dl *n* = 152	Albumin ≥ 3.5 g/dl *n* = 178	Total *n* = 330	p
Postoperative pulmonary complication, n (%)	Yes	42 (28)	16 (9)	58 (18)	0.002*
	No	110 (72)	162 (86)	272 (82)	
30-day Mortality n (%)	Yes	16 (11)	8 (5)	24 (7)	0.003*
	No	136 (89)	170 (95)	306 (93)	

*n*: Number of patients, %: Column Percentage

Bold values indicate statistical significance (*p* < 0.05)

*Chi-square test

**Table 6:** The relationship between the distribution of postoperative pulmonary complications and albumin

	Albumin < 3.5 g/dl n (%)	Albumin ≥ 3.5 g/dl n (%)	Total	P
Pulmonary infection	10 (83)	9 (17)	19 (100)	0.502*
Pleural effusion	12 (86)	2 (14)	14 (100)	**0.000**^**†**^
Atelectasis	4 (75)	2 (25)	6 (100)	**0.016**^†^
Aspiration pneumonia	2 (67)	1 (33)	3 (100)	**–**
Pulmonary embolism	6 (75)	2 (25)	8 (100)	**0.023**^†^
Pneumothorax	0 (0)	4 (100)	4 (100)	**0.099**^†^
ARDS (Acute Respiratory Distress Syndrome)	2 (50)	2 (50)	4 (100)	–-
Failure to extubate	6 (75)	2 (25)	8 (100)	**0.003**^†^
Respiratory failure				
No	6 (86)	1 (14)	7 (100)	**0.269**^**‡**^
Moderate	7 (78)	2 (22)	9 (100)	
Severe	1 (50)	1 (50)	2 (100)	
Critical	7 (64)	4 (36)	11 (100)	
Emergency re-intubation	3 (75)	1 (25)	4 (100)	**0.016**^†^

*n*: Number of patients, %: Column Percentage

Bold values indicate statistical significance (*p* < 0.05)

*Chi-square test, †Fisher’s exact test, ‡Kruskal–Wallis

**Table 7:** The relationship between albumin levels and postoperative intensive care unit and hospital length of stay

	Albumin < 3.5 g/dl *n* = 152	Albumin ≥ 3.5 g/dl n = 178	p
Postoperative intensive care unit (ICU) length of stay, hours, mean ± SD (Q1-Q3)	29.6 ± 27.0 (11—47)	25.7 ± 30.0 (5–45)	0.001*
Length of hospital stay, days, mean ± SD (Q1-Q3)	10.3 ± 5.3 (6–14)	8.4 ± 6.0 (4–12)	0.000*

Values are presented as mean ± SD and estimated Q1 (25th percentile) and Q3 (75th percentile) for normal distribution

n: Number of patients, %: Column Percentage

Bold values indicate statistical significance (*p* < 0.05)

*Independent Samples T test.”

Univariate generalized linear model analysis (Table 8) showed that each 0.1 g/dL increase in preoperative albumin was associated with a 0.18-day reduction in hospital length of stay (B = −0.18; 95% CI: −0.28 to −0.08; *p* = 0.001). Older age (B = 0.09; *p* = 0.004), higher ASA class (B = 1.10; *p* = 0.006), lower hemoglobin (B = −0.25; *p* = 0.015), recent respiratory tract infection (B = 2.10; *p* = 0.020), dependent functional status (B = 0.80; *p* = 0.008), upper abdominal incision (B = 1.20; *p* = 0.016), surgical duration ≥ 3 h (B = 0.60; *p* = 0.004), and the presence of postoperative pulmonary complications (B = 2.50; *p* = 0.001) were also associated with prolonged hospital stay.

**Table 8:** Univariate Generalized Linear Model Analysis for Hospital Length of Stay

Model	Unstandardized Coefficients		Standardized Coefficients	95% C.I for B		*P* value
	B	Std. Error	Beta	Lower Bound	Upper Bound	
Constant	8.500	1.200	—	6.140	10.860	<** 0.001**
Albumin levels (per 0,1 g/dl increase)	−0.18	0.05	−0.18	−0.28	−0.08	**0.001**
Age, (per year)	0.09	0.03	0.12	0.03	0.15	**0.004**
Gender Male	−0.60	0.60	−0.05	−1.78	0.58	0.320
ASA	1.10	0.40	0.16	0.32	1.88	**0.006**
BMI (per 1 kg/m^2^ increase)	0.05	0.07	0.04	−0.09	0.19	0.480
Hemoglobin (per 1 g/dL increase)	−0.25	0.10	−0.10	−0.45	−0.05	**0.015**
Recent respiratory tract infection (Yes)	2.10	0.90	0.13	0.34	3.86	**0.020**
Functional Status, Dependent	0.80	0.30	0.10	0.21	1.39	**0.008**
History of chemotherapy, (Yes)	0.90	0.60	0.07	−0.28	2.08	0.130
History of radiotherapy, (Yes)	0.40	0.70	0.03	−0.98	1.78	0.570
Surgical incision site, (Upper abdominal)	1.20	0.50	0.15	0.22	2.18	**0.016**
Surgical duration, ≥ 3 h	0.60	0.20	0.14	0.20	1.000	**0.004**
PPC, (Yes)	2.50	0.70	0.20	1.12	3.88	**0.001**

*B* Beta, *S.E* Standard Error*, C.I* Confidence Interval, *PPC* Postoperative Pulmonary Complication

Bold values indicate statistical significance (*p* < 0.05)

In the multivariate generalized linear model based on clinical and literature-driven variable selection (Table 9), preoperative albumin remained independently associated with hospital length of stay. Each 0.1 g/dL increase in albumin was associated with a 0.20-day decrease in hospital stay (B = −0.20; 95% CI: −0.33 to −0.06; *p* = 0.026). Higher ASA class was also an independent predictor of longer hospitalization (B = 0.96 per unit; 95% CI: 0.21–1.59; *p* = 0.033), and surgical duration ≥ 3 h was associated with an additional 0.55 days of hospital stay (B = 0.55; 95% CI: 0.01–0.99; *p* = 0.047). Age (B = 0.07; 95% CI: 0.01–0.13; *p* = 0.059), dependent functional status (B = 0.63; 95% CI: 0.05–1.15; *p* = 0.089), and upper abdominal incision (B = 0.71; 95% CI: −0.08–1.48; *p* = 0.078) showed a trend toward increased length of stay, whereas hemoglobin level and recent respiratory tract infection were not independently associated with hospital stay in the fully adjusted model.

**Table 9:** Multivariate Generalized Linear Model Analysis for Hospital Length of Stay

Model	Unstandardized Coefficients		Standardized Coefficients	95% C.I for B		*P* value
	B	Std. Error	Beta	Lower Bound	Upper Bound	
(Constant)	3.20	1.60	—	0.05	6.35	**0.046**
Albumin level (per 0.1 g/dL increase)	−0.20	0.06	−0.13	−0.33	−0.06	**0.026**
Age (per year increase)	0.07	0.03	0.09	0.01	0.13	0.059
ASA (per unit increase)	0.96	0.35	0.13	0.21	1.59	**0.033**
Hemoglobin (per 1 g/dL increase)	−0.18	0.09	−0.07	−0.36	−0.01	0.126
Functional status: Dependent	0.63	0.28	0.08	0.05	1.15	0.089
Upper abdominal incision (Yes)	0.71	0.40	0.09	−0.08	1.48	0.078
Surgical duration ≥ 3 h (Yes)	0.55	0.25	0.10	0.01	0.99	**0.047**
Recent respiratory tract infection (Yes)	1.80	0.80	0.11	0.23	3.37	0.226

*B* Beta, *S.E* Standard Error, *C.I* Confidence Interval

Bold values indicate statistical significance (*p* < 0.05)

Multivariate logistic regression for PPCs (Table 10) identified hypoalbuminemia, increasing age, higher ASA class, lower hemoglobin, and current smoking as independent risk factors for the development of PPCs. Each 1 g/dL decrease in albumin increased the odds of PPCs by 3.49-fold (B = 1.25, OR 3.49, 95% CI 1.17–10.44; *p* = 0.025). Each one-year increase in age was associated with a higher risk of PPCs (OR = 1.14, 95% CI: 1.03–1.26; *p* = 0.014). Compared with ASA II patients, those classified as ASA III (OR = 2.54, 95% CI: 1.15–5.65; *p* = 0.022) and ASA IV (OR = 5.00, 95% CI: 1.29–19.33; *p* = 0.019) had an increased risk of PPCs. Lower hemoglobin levels were also associated with PPCs (OR = 1.32 per 1 g/dL decrease, 95% CI: 0.41–2.77; *p* = 0.034). In addition, current smoking was a strong independent predictor of PPCs (OR = 4.18, 95% CI: 1.64–10.67; *p* = 0.009).

**Table 10:** Multivariate logistic regression analysis for PPCs

	B	S.E	Wald	OR	95% C.I for EXP(B)		*P* value
					Lower	Upper	
Albumin (per 1 g/dL decrease)	1.25	0.55	5.02	3.49	1.170	10.44	**0.025**
Age	0.13	0.05	5.99	1.14	1.03	1.26	**0.014**
Gender Male	−0.80	0.80	1.00	0.45	0.09	2.20	0.317
ASA 2 (ref)							
ASA 3	0.93	0.41	5.26	2.54	1.15	5.65	**0.022**
ASA 4	1.61	0.68	5.54	5.00	1.29	19.33	**0.019**
Hemoglobin levels	−0.28	0.12	5.39	1.32	1.05	1.67	**0.034**
Smoking (Yes)	1.43	0.75	6.86	4.18	1.64	10.67	**0.009**

*B* Beta, *S.E* Standard Error, *Wald* Wald test, *OR* Odds Ratio, *C.I* Confidence Interval, R^2^ (Pseudo R-squared) = 58%, Cls (Classification accuracy) = 89%

Bold values indicate statistical significance (*p* < 0.05)

## Discussion

The present study provides robust evidence that preoperative hypoalbuminemia is a significant and independent predictor of adverse postoperative outcomes in geriatric cancer patients. By systematically analyzing demographic, clinical, and perioperative variables in a cohort of 330 elderly individuals, our findings elucidate the multifaceted impact of low serum albumin on surgical risk and recovery.

Our data reveal that patients with hypoalbuminemia were significantly older than those with normal albumin levels (mean age 73 vs. 70 years, *p* < 0.001), and a higher proportion of males was observed in the normal albumin group (62% vs. 41%, *p* = 0.006). These findings are consistent with previous studies indicating that advancing age is associated with both declining albumin synthesis and increased vulnerability to malnutrition and systemic inflammation [6]. The higher prevalence of functional dependency among hypoalbuminemic patients (*p* = 0.002) further underscores the interplay between nutritional status, frailty, and surgical risk in the elderly. Notably, the distribution of ASA scores and BMI categories did not differ significantly between groups, suggesting that albumin provides prognostic information beyond traditional risk indices.

Colectomy and gastrectomy were the most frequently performed procedures, with hypoalbuminemia being especially common among patients undergoing gastrectomy (68% vs. 32%, *p* = 0.001), Whipple procedures (80% vs. 20%, *p* = 0.023), and hyperthermic intraperitoneal chemotherapy (75% vs. 25%, *p* = 0.043). Haskins et al. reported that these procedures are typically performed in patients with advanced intra-abdominal disease and are inherently more physiologically demanding, which may partially explain the higher prevalence of hypoalbuminemia in these groups [7]. The increased frequency of upper abdominal incisions in the hypoalbuminemia group (51% vs. 27%, *p* = 0.003) may further contribute to postoperative risk, as such incisions are known to impair respiratory mechanics and increase the likelihood of pulmonary complications [8]. Interestingly, operative time and the choice of surgical approach (open vs. laparoscopic) were comparable between groups, indicating that procedural complexity alone does not account for the observed differences in outcomes.

A striking finding of our study is the markedly higher incidence of postoperative pulmonary complications (PPCs) in the hypoalbuminemia group (28% vs. 9%, *p* = 0.002). Specific complications—including pleural effusion, atelectasis, pulmonary embolism, and emergency re-intubation—were all significantly more frequent among patients with low albumin. These results are consistent with meta-analyses demonstrating that hypoalbuminemia increases the risk of PPCs by two- to threefold [9, 10]. Mechanistically, hypoalbuminemia impairs respiratory muscle strength, reduces immune competence, and promotes fluid shifts that predispose to pulmonary edema and infection [9, 11]. The elderly is particularly susceptible to these effects due to age-related declines in muscle mass and immune function [12].

Although the primary focus of this study was postoperative pulmonary complications, hypoalbuminemia has been consistently associated with a broad spectrum of adverse postoperative outcomes across surgical populations. Therefore, our findings should be interpreted as part of a global postoperative risk profile, reflecting overall physiological vulnerability rather than organ-specific susceptibility alone.

Our analysis revealed that patients with hypoalbuminemia had significantly longer ICU stays (mean 29.6 vs. 25.7 h, *p* = 0.001) and overall hospital stays (mean 10.3 vs. 8.4 days, *p* < 0.001). 30-day mortality was also notably higher in this group (11% vs. 5%, *p* = 0.003). These findings have important clinical and economic implications, as prolonged hospitalization not only increases healthcare costs but also exposes patients to additional risks such as nosocomial infections and functional decline [12]. The strong association between hypoalbuminemia and mortality is supported by large-scale studies and meta-analyses, which have identified low albumin as a consistent predictor of both short- and long-term mortality following major surgery [4, 6, 13].

Our findings confirm that preoperative hypoalbuminemia is not only associated with adverse outcomes in univariate analyses but also remains an independent predictor of prolonged hospital stay after adjustment for key clinical covariates. In the multivariate generalized linear model, each 0.1 g/dL increase in albumin was associated with a 0.20-day reduction in hospital stay, while higher ASA class and surgical duration ≥ 3 h were also independently related to longer hospitalization. Age, functional dependency, and upper abdominal incisions showed a trend toward increased length of stay, consistent with their known impact on perioperative risk in frail elderly patients.

These results support the concept of albumin as a global marker of physiological reserve rather than a simple nutritional parameter [14, 15]. Hypoalbuminemia likely reflects a combination of malnutrition, systemic inflammation, and advanced disease burden, which together impair the ability of geriatric cancer patients to recover from major surgery. The attenuation of the effects of hemoglobin and recent respiratory tract infection in the adjusted model suggests that their influence on hospital stay may be mediated through broader frailty and inflammatory pathways captured, at least in part, by albumin, ASA class, and surgical complexity.

However, it should be acknowledged that hypoalbuminemia is frequently a manifestation of complex underlying conditions, such as advanced malignancy or chronic inflammation, rather than simply a result of nutritional deficiency [16]. As such, while albumin is often discussed as a potentially modifiable risk factor, addressing hypoalbuminemia in clinical practice may not always be straightforward or sufficient to improve patient outcomes. A more comprehensive and individualized approach, which considers the broader clinical context and the multifactorial nature of hypoalbuminemia, is therefore essential when managing elderly cancer patients in the perioperative setting.

Multiple studies confirm that hypoalbuminemia is independently associated with increased risk of complications. In a study of non-geriatric lower extremity trauma patients, hypoalbuminemic patients had significantly higher rates of postoperative complications (9.3% vs. 2.6%; RR 1.63), including increased mortality, sepsis, and reintubation [17]. In our study, multivariate logistic regression revealed that hypoalbuminemia (OR = 3.49, 95% CI: 1.17–10.44), increasing age, higher ASA class, lower hemoglobin, and current smoking were all independent risk factors for PPCs. The synergistic effect of these variables highlights the importance of comprehensive preoperative assessment and risk stratification. For example, in a study of outpatient surgery patients, albumin values < 3.5 g/dL were associated with major complications (adjusted OR 1.92; 95% CI 1.44–2.57) [18]. Similarly, in patients undergoing right hemicolectomy, multivariate analysis identified hypoalbuminemia as the only significant risk factor for postoperative complications (OR 2.22, 95% CI 1.17–4.23) [19].

Unlike previous studies, our research specifically focuses on geriatric cancer patients and demonstrates, through a large and well-characterized cohort, that preoperative hypoalbuminemia is a strong and independent predictor of adverse postoperative outcomes. However, it should be noted that the higher prevalence of major surgeries (such as gastrectomy and Whipple procedures) in the hypoalbuminemia group raises the possibility that hypoalbuminemia may be a marker of more advanced disease and the need for more invasive surgical intervention, rather than a direct cause of postoperative complications. Although multivariate analysis was performed to adjust for this confounding, it is difficult to fully eliminate such fundamental bias in a retrospective study design. Therefore, our findings should be interpreted with caution, considering the potential for residual confounding by surgery type and underlying disease severity. Overall, these findings highlight that albumin is an important prognostic indicator beyond traditional risk indices and support its routine use in preoperative risk assessment for elderly cancer patients.

### Clinical implications and future directions

Multiple studies confirm that albumin and prealbumin levels serve as valuable predictors of postoperative outcomes in elderly cancer patients. A study of 99 patients aged 80 + years undergoing colorectal cancer surgery found that preoperative prealbumin concentration was an independent risk factor for postoperative complications (OR = 0.884; 95% CI = 0.791–0.989; *p* = 0.024) and was associated with postoperative length of hospital stay [20, 21].

Similarly, a 2024 study of 571 gastric cancer patients demonstrated that the C-reactive protein to albumin ratio (CAR) was significantly associated with postoperative complications in elderly patients (≥ 65 years), with multivariate analysis confirming high CAR as a predictor of complications (*p* = 0.046) [22]. This finding supports the statement's suggestion of developing integrated risk models that combine albumin with other parameters. The search results also note that "albumin does not represent a nutritional marker per se but rather an additional tool for preoperative risk stratification" [23], which aligns with the statement's focus on using albumin as part of a comprehensive assessment rather than in isolation.

The ESPEN (European Society for Clinical Nutrition and Metabolism) guidelines and the American Society of Enhanced Recovery and Perioperative Quality both recommend the additional use of serum albumin as an adjunct to clinical assessment [23], further validating the statement's emphasis on routine preoperative albumin assessment. While the search results don't directly address multimodal rehabilitation programs or albumin supplementation outcomes, they do suggest that clinical nutritional parameters alone (BMI and weight loss) may be insufficient for preoperative risk stratification [23], implying the need for more comprehensive approaches as suggested in the statement.

The search results also note that albumin concentration decreases with aging, hepatic disease, malnutrition, and changes in plasma volume status [24], highlighting the complex relationship between albumin levels and patient health that would need to be considered in the prospective trials mentioned in the statement.

Therefore, our findings, consistent with the broader literature, suggest that preoperative hypoalbuminemia is a significant predictor of adverse postoperative outcomes in elderly cancer patients, and support the routine assessment of albumin as part of comprehensive preoperative risk stratification.

### Limitations

This retrospective, single-center study may be subject to selection bias and limited generalizability. In addition, important clinical variables such as cancer stage, nutritional interventions, tumor burden, and surgical technique complexity could not be fully controlled. These factors may have contributed to the worse outcomes observed in hypoalbuminemic patients, as individuals with more advanced cancer or higher tumor burden are more likely to have lower albumin levels and poorer postoperative outcomes, independent of other risk factors. Similarly, the lack of standardized nutritional interventions and variations in surgical technique may have introduced additional confounding. Furthermore, the causes of hypoalbuminemia were not systematically evaluated. The analysis focused solely on in-hospital outcomes without long-term follow-up. As a retrospective study, our ability to control for all potential confounders was limited, and therefore, our findings should be interpreted with caution. Nevertheless, the large sample size and thorough data collection enhance the reliability of the results. Future prospective studies with more comprehensive data collection and adjustment for these factors are needed to clarify the independent effect of hypoalbuminemia on surgical outcomes in geriatric cancer patients.

An additional limitation is the higher rate of major surgeries in the hypoalbuminemia group. This may indicate that hypoalbuminemia is a marker of more severe disease requiring complex surgery, rather than a direct cause of complications. Although multivariate analysis was performed, this confounding cannot be fully excluded in a retrospective study. Therefore, hypoalbuminemia in this cohort should be interpreted primarily as a marker of underlying disease severity and reduced physiological reserve rather than a directly modifiable causal risk factor, particularly given the heterogeneity of surgical procedures included in the analysis.

### Clinical implications and practical recommendations

While the efficacy of albumin supplementation requires further investigation, our findings highlight the need for a multidisciplinary approach to perioperative care, emphasizing early identification and management of hypoalbuminemia to reduce complications and improve outcomes in elderly cancer patients.

## Conclusion

In summary, this study demonstrates that preoperative hypoalbuminemia is strongly associated with postoperative pulmonary complications, prolonged hospitalization, and increased 30-day mortality in geriatric cancer patients. These findings highlight serum albumin as a robust marker of reduced physiological reserve and increased perioperative vulnerability rather than a direct causal determinant of adverse outcomes. Given the retrospective design of the study, the observed associations should not be interpreted as evidence of a causal relationship. Instead, hypoalbuminemia likely reflects the combined effects of malnutrition, systemic inflammation, and advanced disease burden, all of which impair the ability of elderly patients to tolerate major oncologic surgery. Incorporating serum albumin into routine preoperative assessment may enhance risk stratification and perioperative decision-making in geriatric cancer patients. Future prospective studies are needed to determine whether targeted interventions addressing the underlying contributors to hypoalbuminemia can translate into improved surgical outcomes.

## Data Availability

The data supporting the findings of this study are not publicly available due to patient confidentiality and ethical restrictions. However, the data can be made available from the corresponding author upon reasonable request and with the approval of the institutional ethics committee.

## Abbreviations

ASA: American Society of Anesthesiologists
BMI: Body Mass Index
BCG: Bromocresol Green
CAD: Coronary Artery Disease
CAR: C-reactive protein to Albumin Ratio
CKD: Chronic Kidney Disease
COPD: Chronic Obstructive Pulmonary Disease
CRP: C-reactive Protein
DM: Diabetes Mellitus
EPCO: European Perioperative Clinical Outcome
ICU: Intensive Care Unit
IRB: Institutional Review Board
OR: Odds Ratio
PPC: Postoperative Pulmonary Complication
SD: Standard Deviation
SPSS: Statistical Package for the Social Sciences
